# Iron on Digestion

**Published:** 1880-08

**Authors:** 


### IRON ON DIGESTION.

Dr. Perry, taking as his subject the *misuse of iron preparations, and their effect upon the digestive process*, supports his views with the following experiments. Two test-tubes containing artificial gastric juice and albumen; into one of them is poured one drachm of elixir of iron and quinia, at the end of four hours digestion has been completed in the tube containing no iron; the albumen remains unaffected in the tube containing the medicine. At the end of ten hours the albumen in the latter is still undigested, showing the effect of the iron preparation in suspending digestion. He quotes cases in which he considers indigestion to have been due to the presence of a preparation of iron in the stomach; he advises that such medicines be adminis tered one hour before, or four hours after meals.

